# EHMTI-0210. Dolotest may reflect the effect of psychological treatment in patients suffering from severe headache

**DOI:** 10.1186/1129-2377-15-S1-D34

**Published:** 2014-09-18

**Authors:** T Zimmer, CB Møller, ML Westergaard, D Kjeldgaard Nielsen

**Affiliations:** 1 Department of Neurology, Danish Headache Center University of Copenhagen, Glostrup, Denmark

## Introduction

Patients with severe headache who undergo psychological treatment at the Danish Headache Center, a tertiary multidisciplinary treatment centre, often report positive effects on their perceived general well-being and ability to work. However there is no agreement on which quality of life (QoL) instruments are useful for monitoring outcome of psychological treatment.

## Aim

To assess the effect of psychological treatment in headache patients using the DOLOtest, a newer validated Health-Related QoL instrument.

## Method

All patients referred to psychological assessment at the Danish Headache Center in 2012 underwent an initial interview and 129 were offered psychological treatment. Treatment was either group treatment or individual therapy. All participants were given the DOLOtest, at baseline, 1st, 5th and last therapy session and at six-month follow-up. DOLOtest-scores were compared using t-test.

## Results

Of the patients offered treatment, 100 accepted, 93 completed, and 71 were reached at six-month follow-up. There was a significant improvement on the following domains at six-month follow-up compared to baseline: pain (p=.039), problems with more strenuous physical activities (p=.037), reduced energy and strength (p<.001), and low spirit (p=.007). Headache frequency was also significantly reduced from 23 to 17 days/month (p<.001).

## Conclusion

The DOLOtest may be a valuable multidimensional instrument to assess QoL in patients with severe headache, and to monitor outcome of psychological treatment in future trials.

No conflict of interest.

